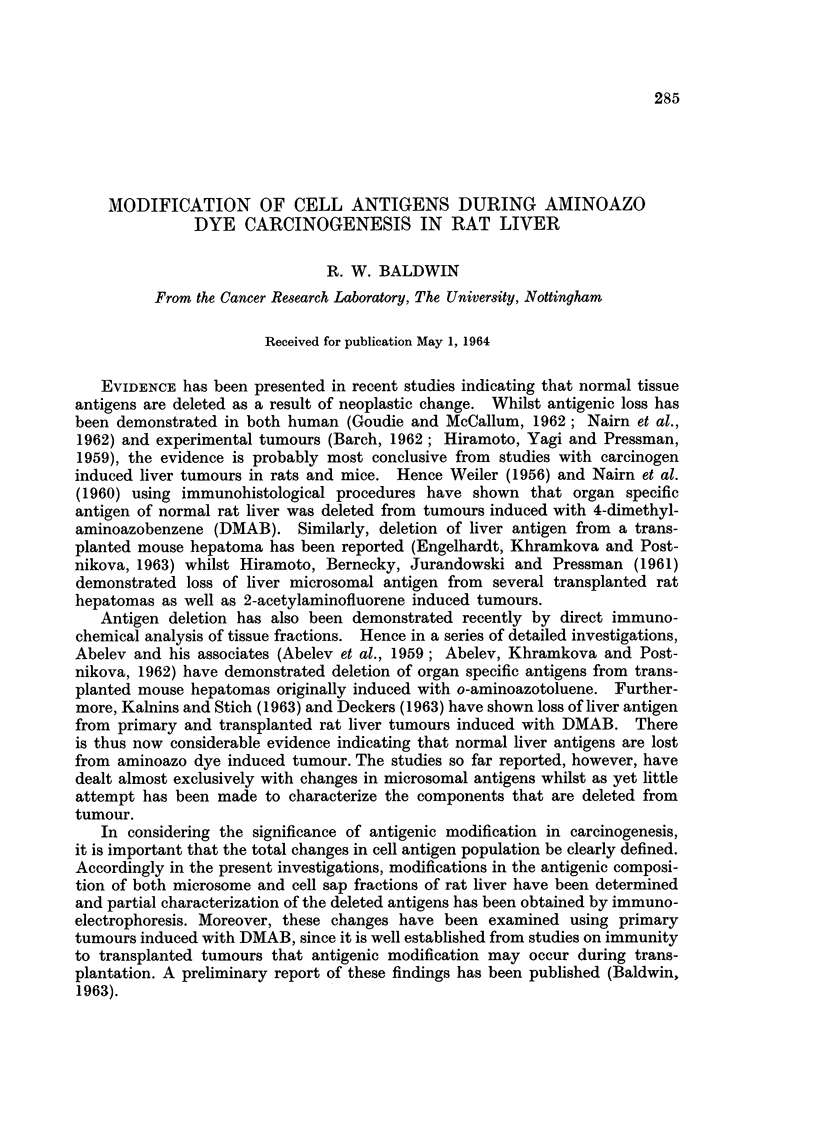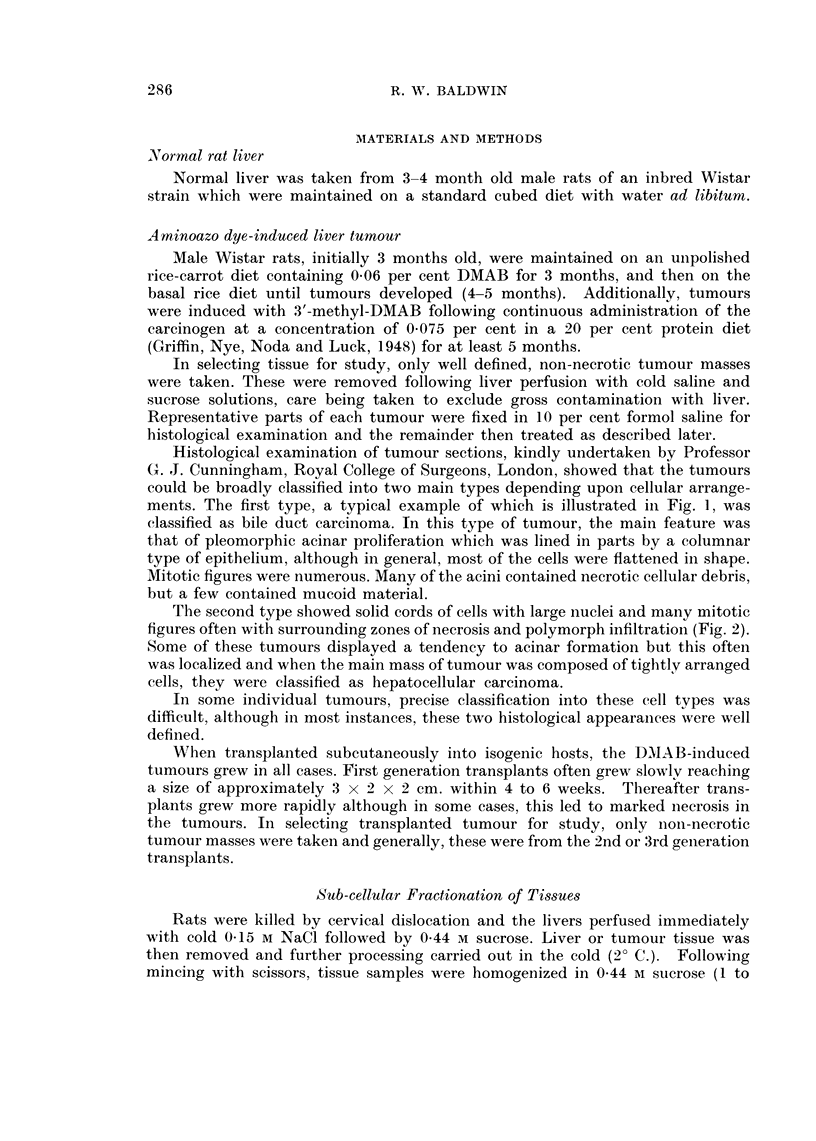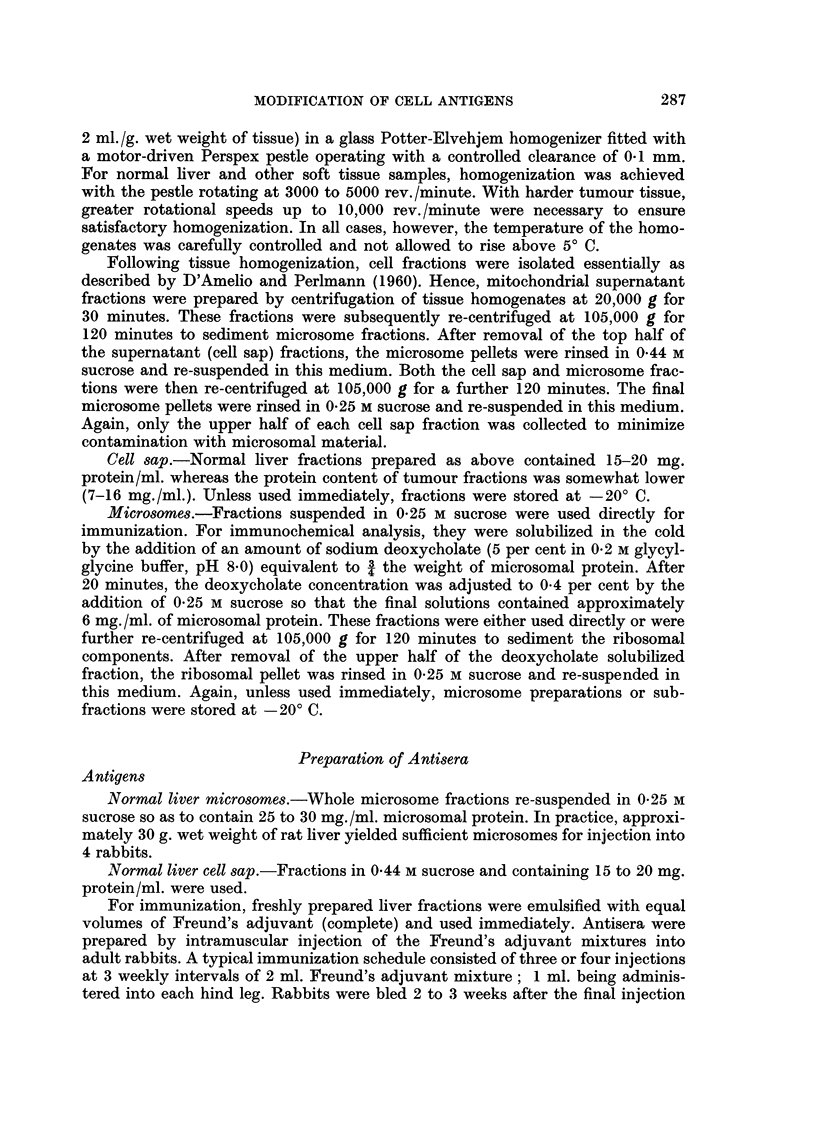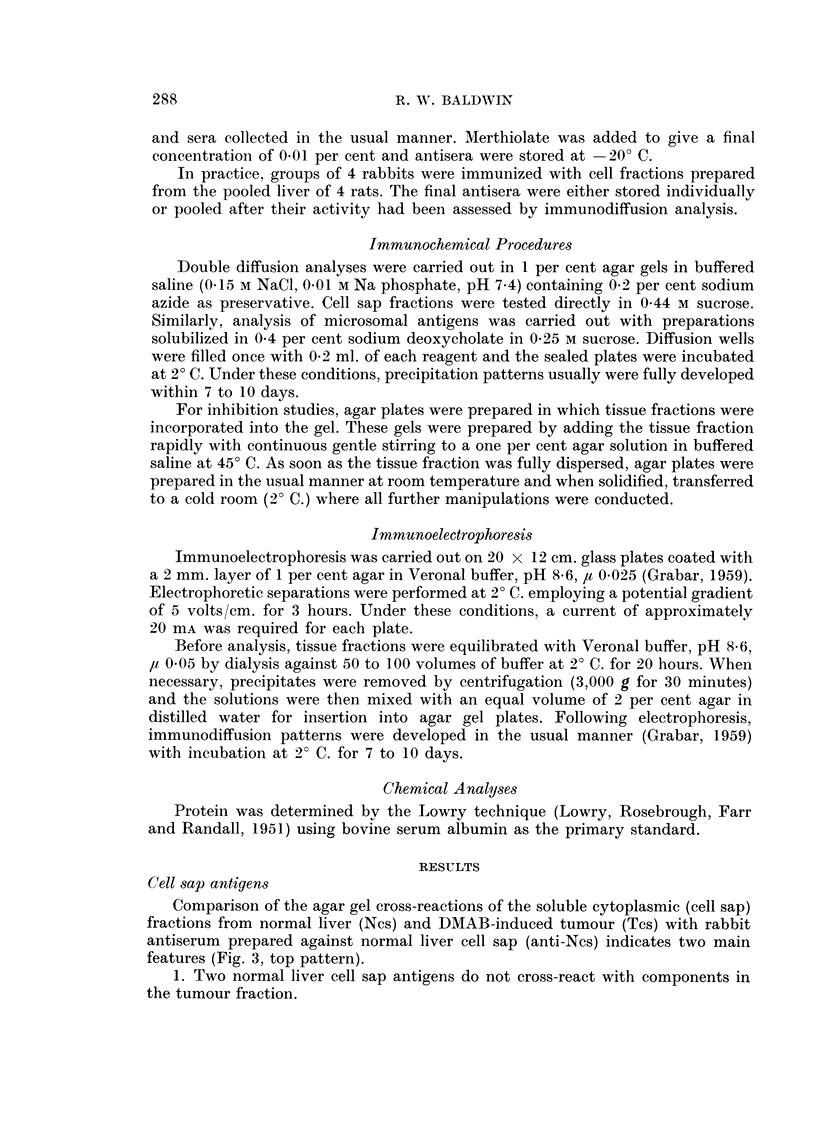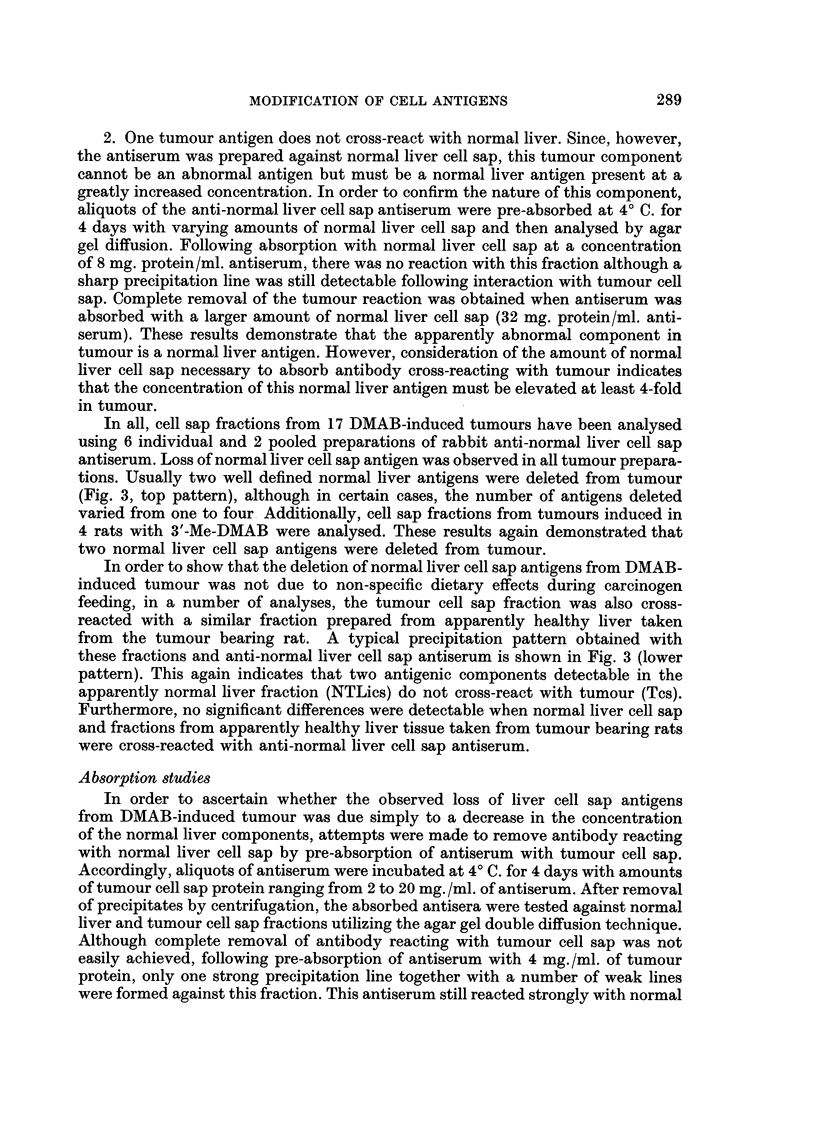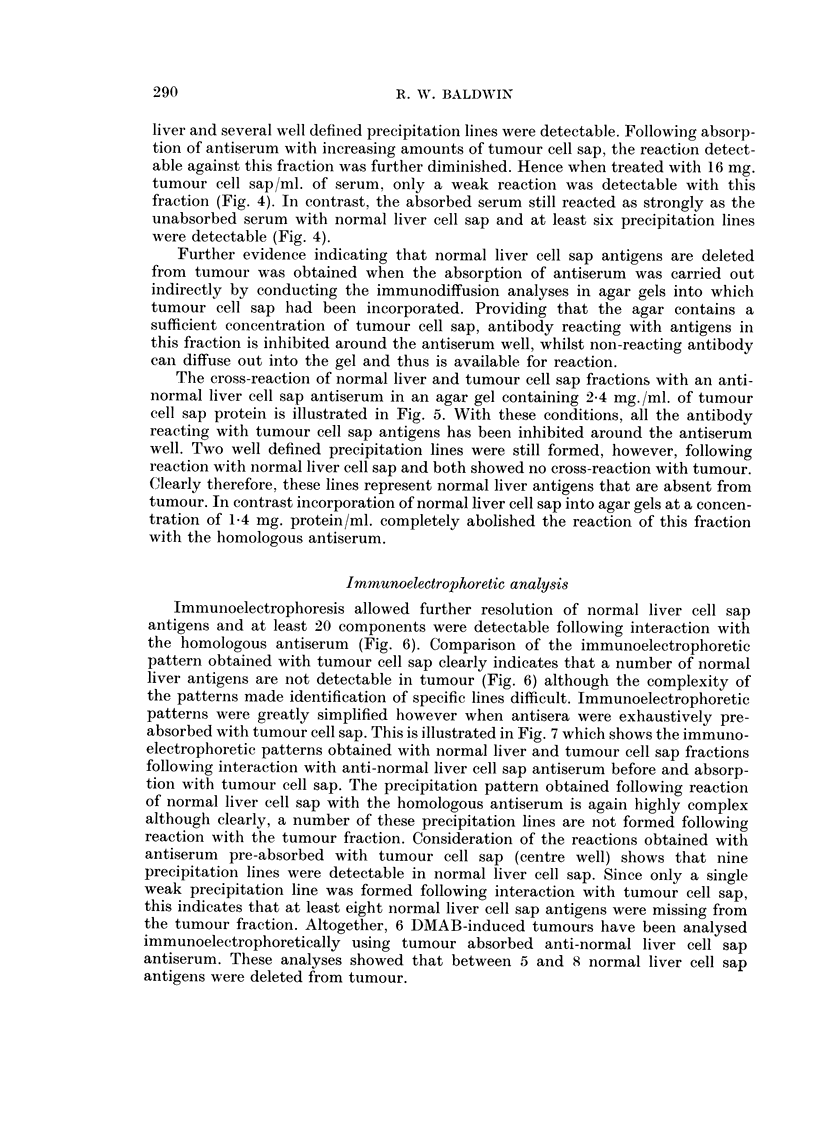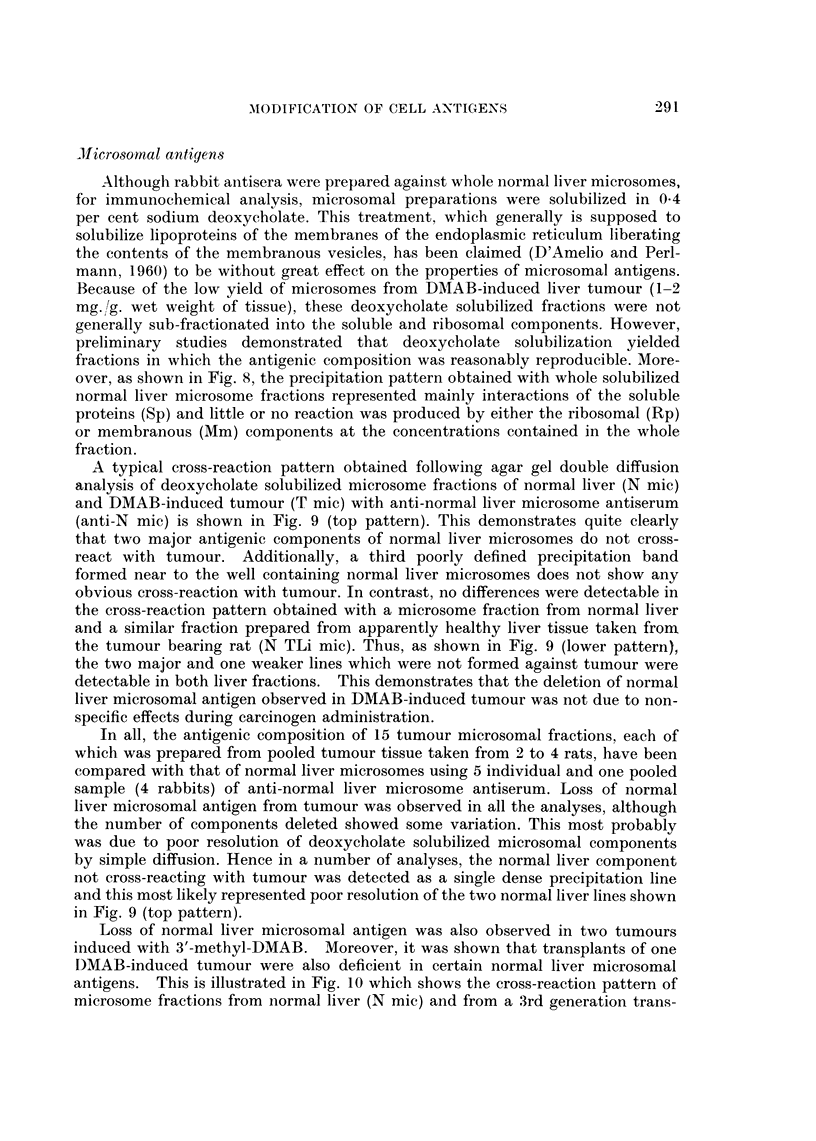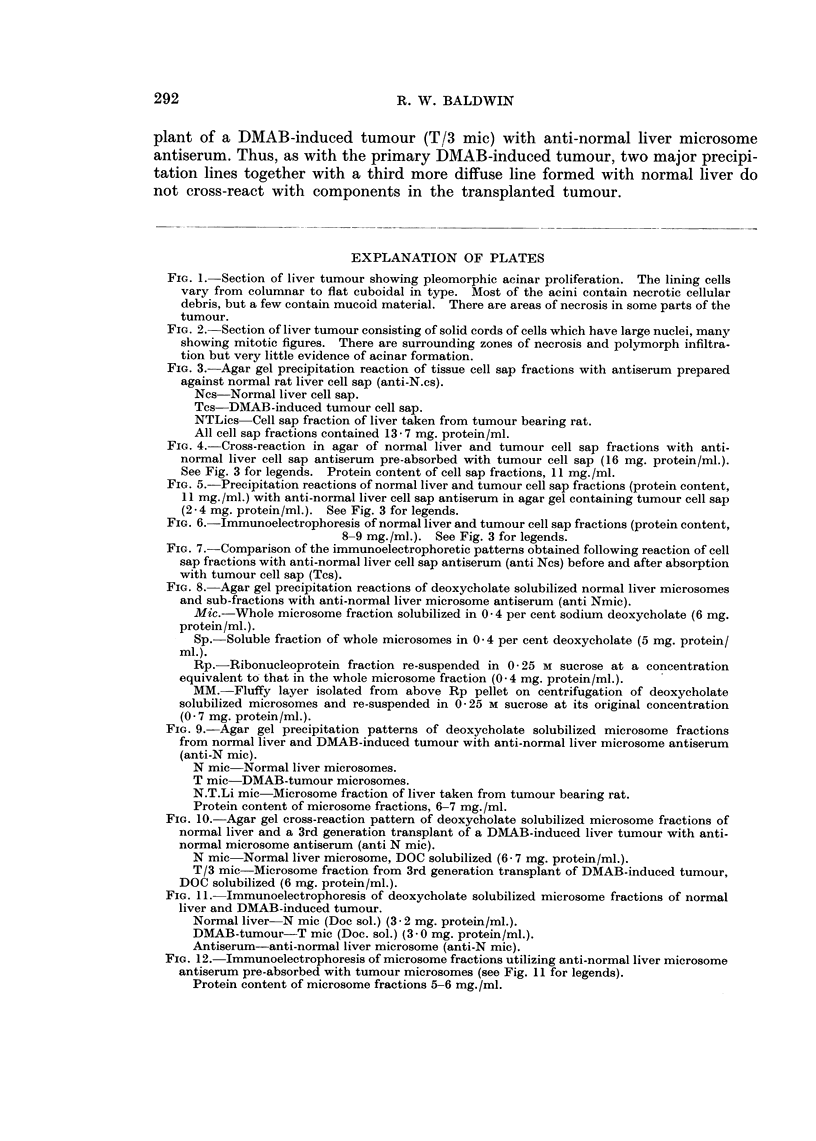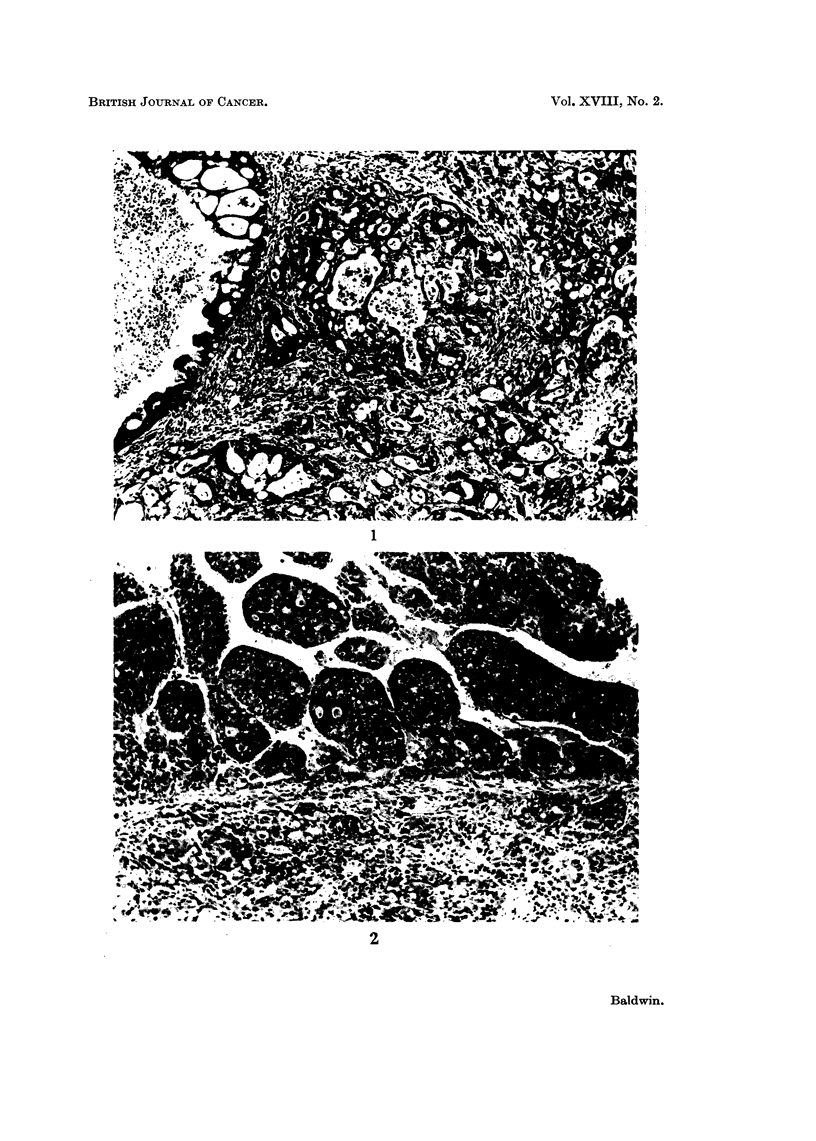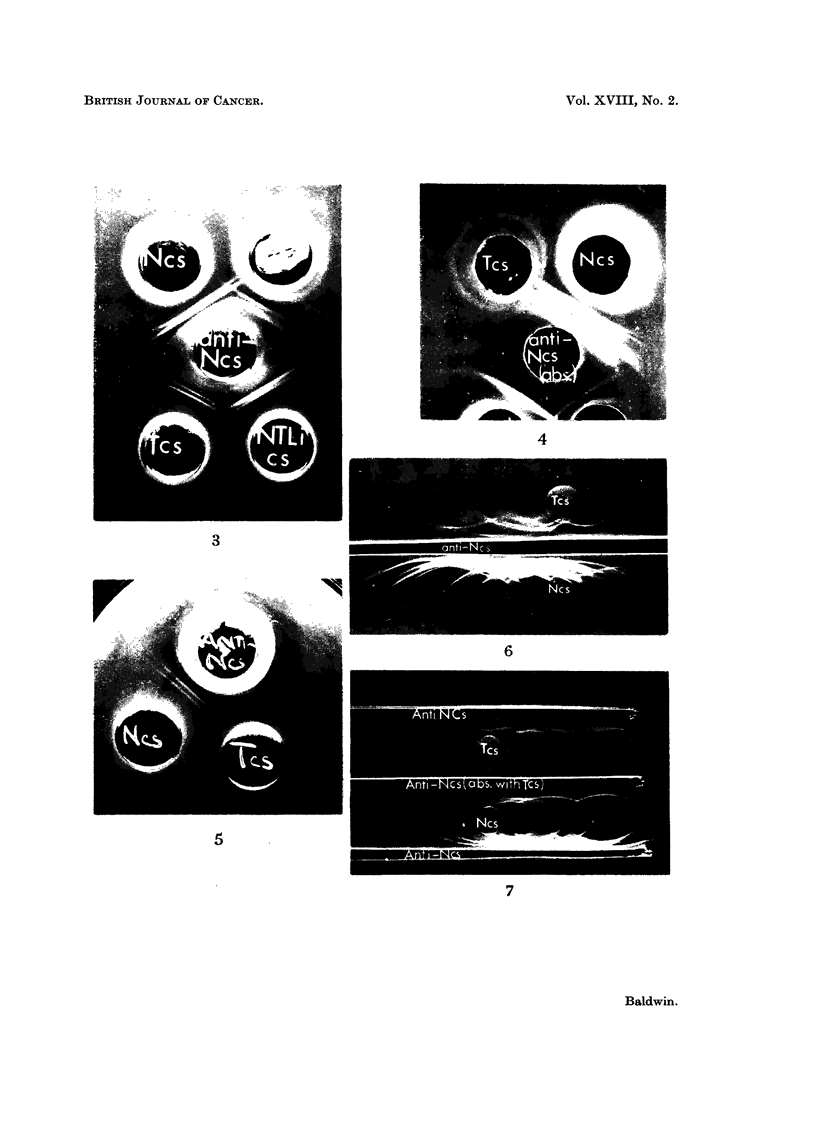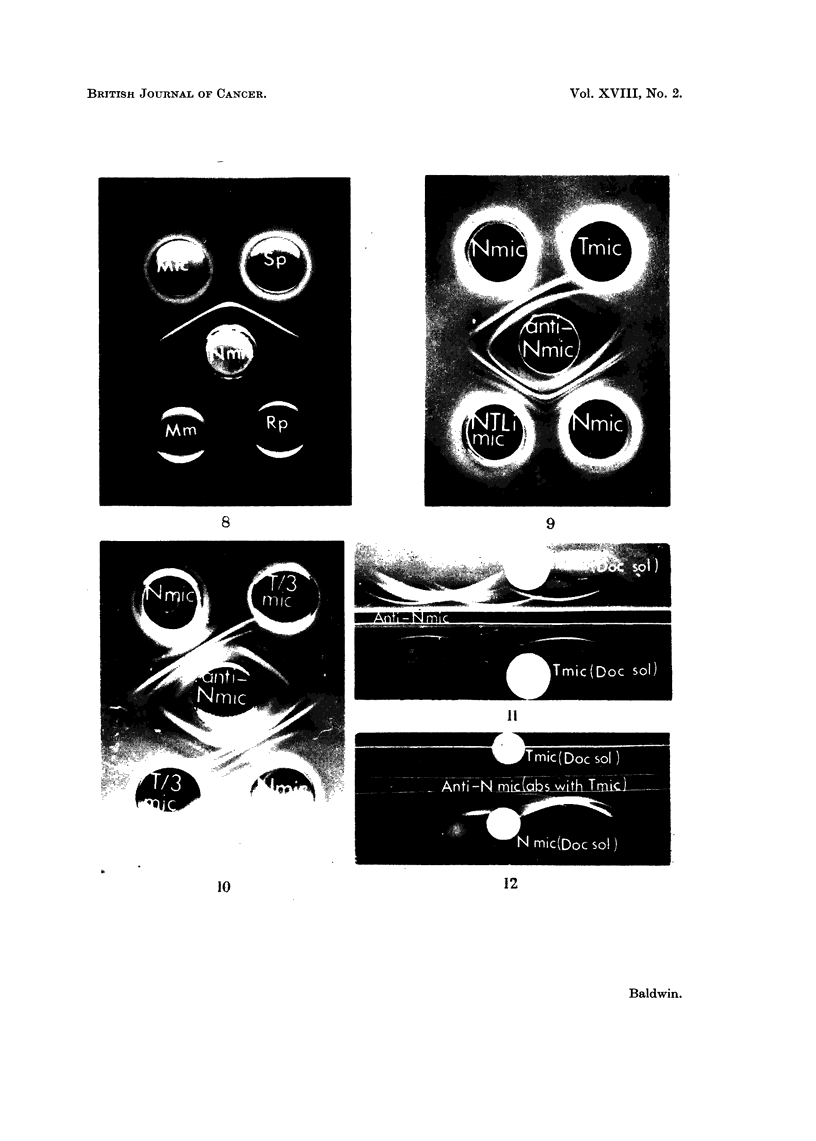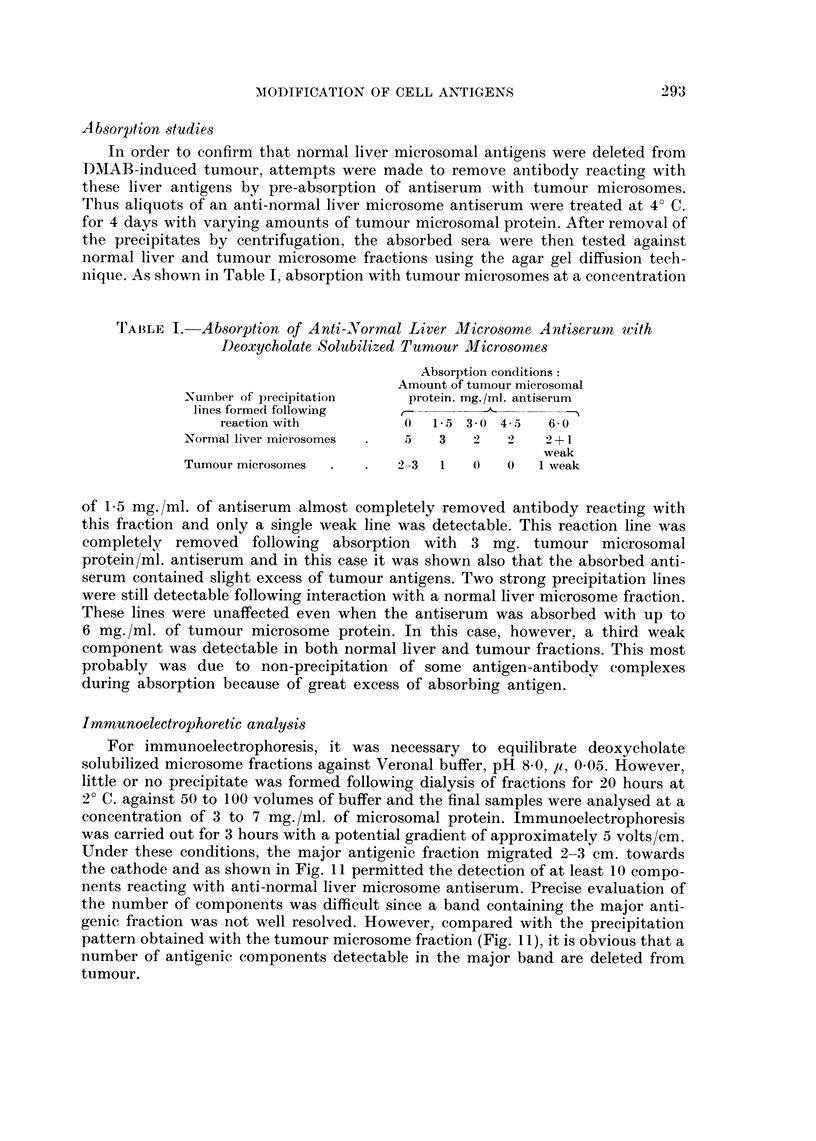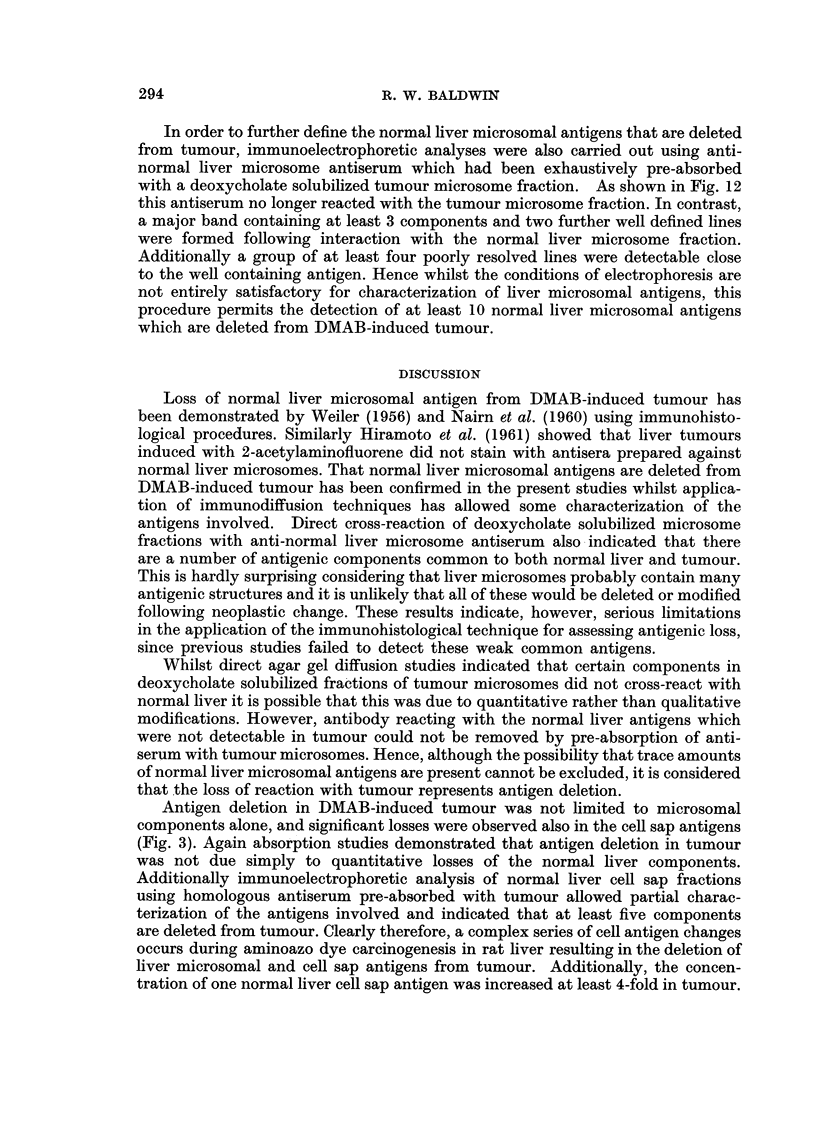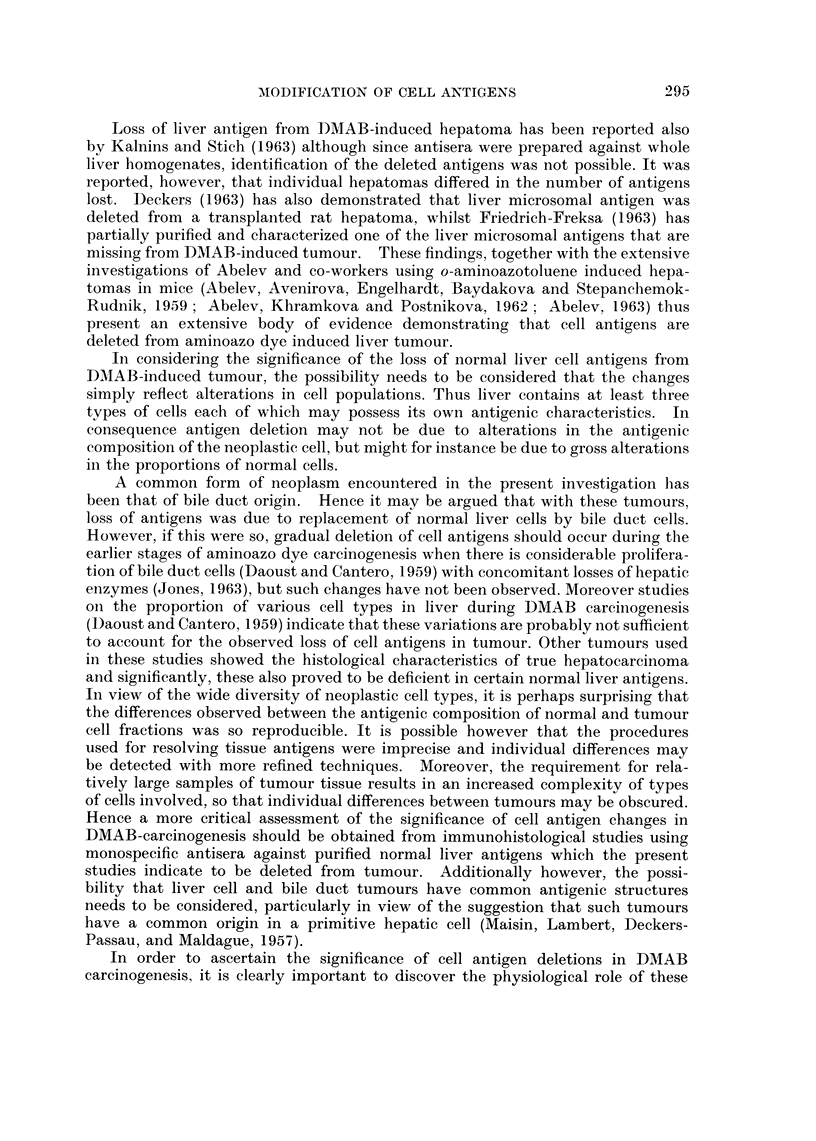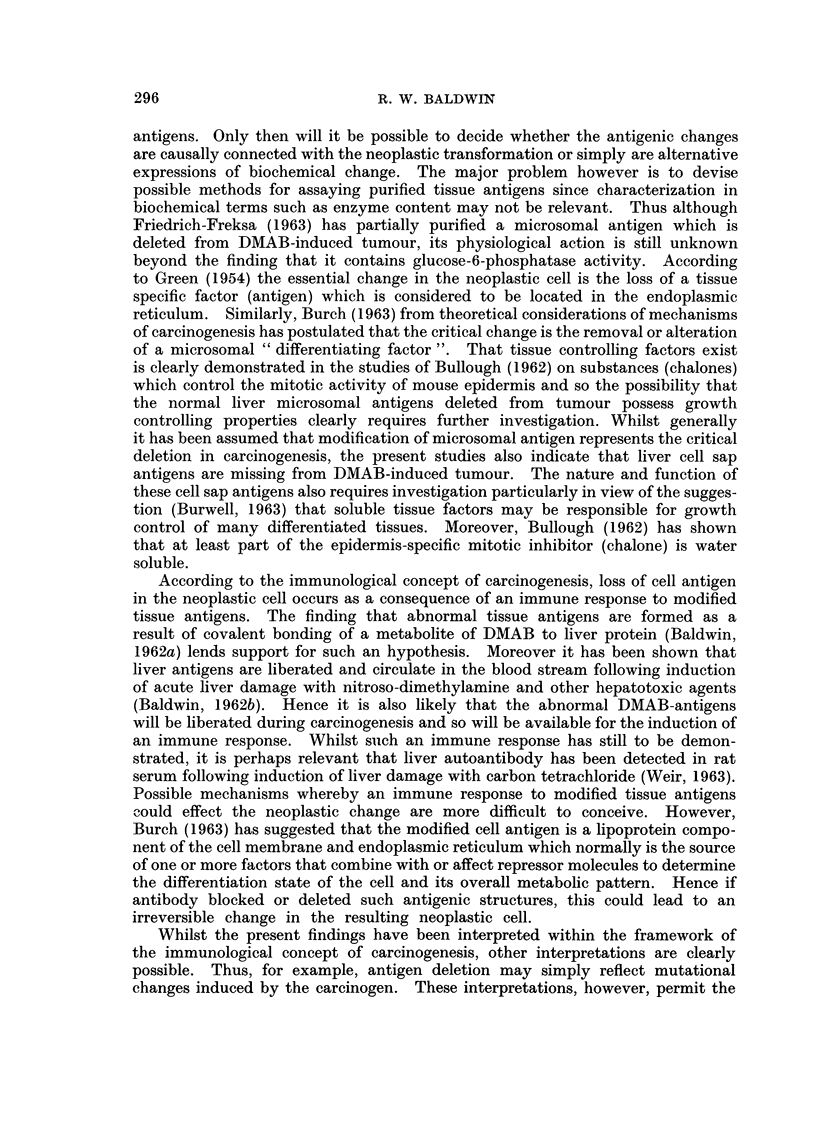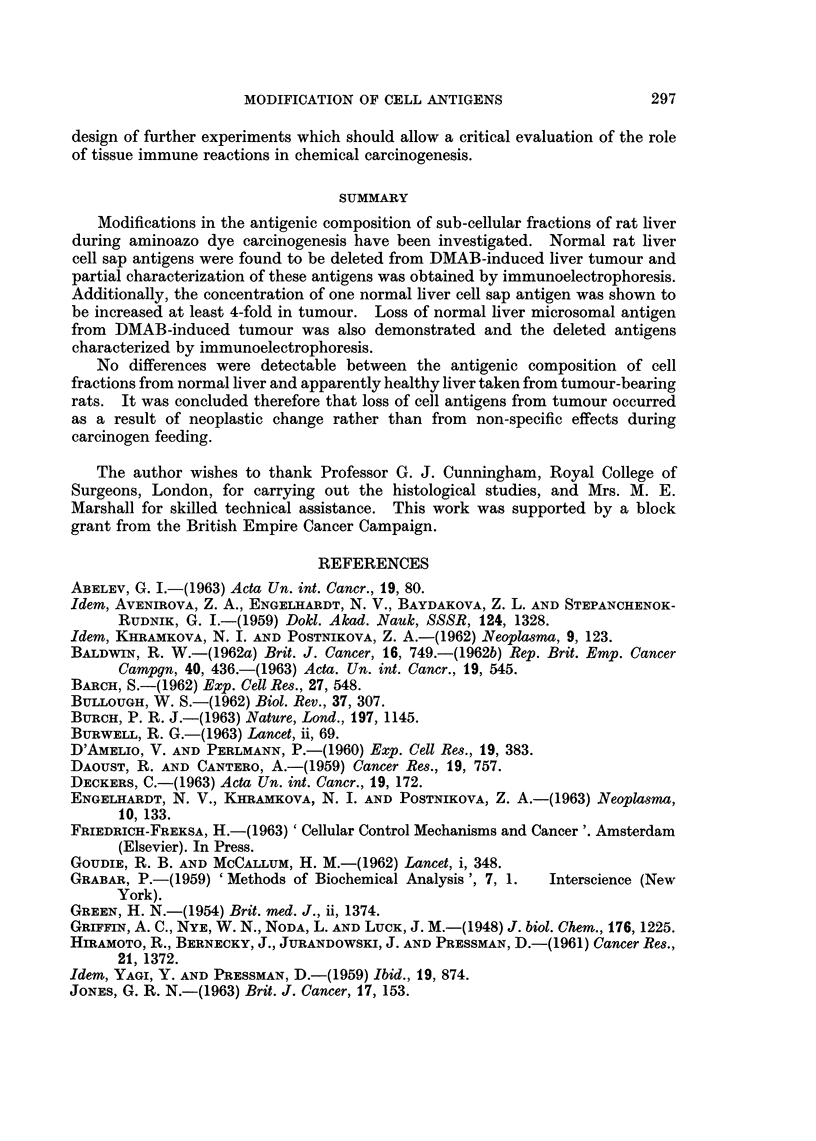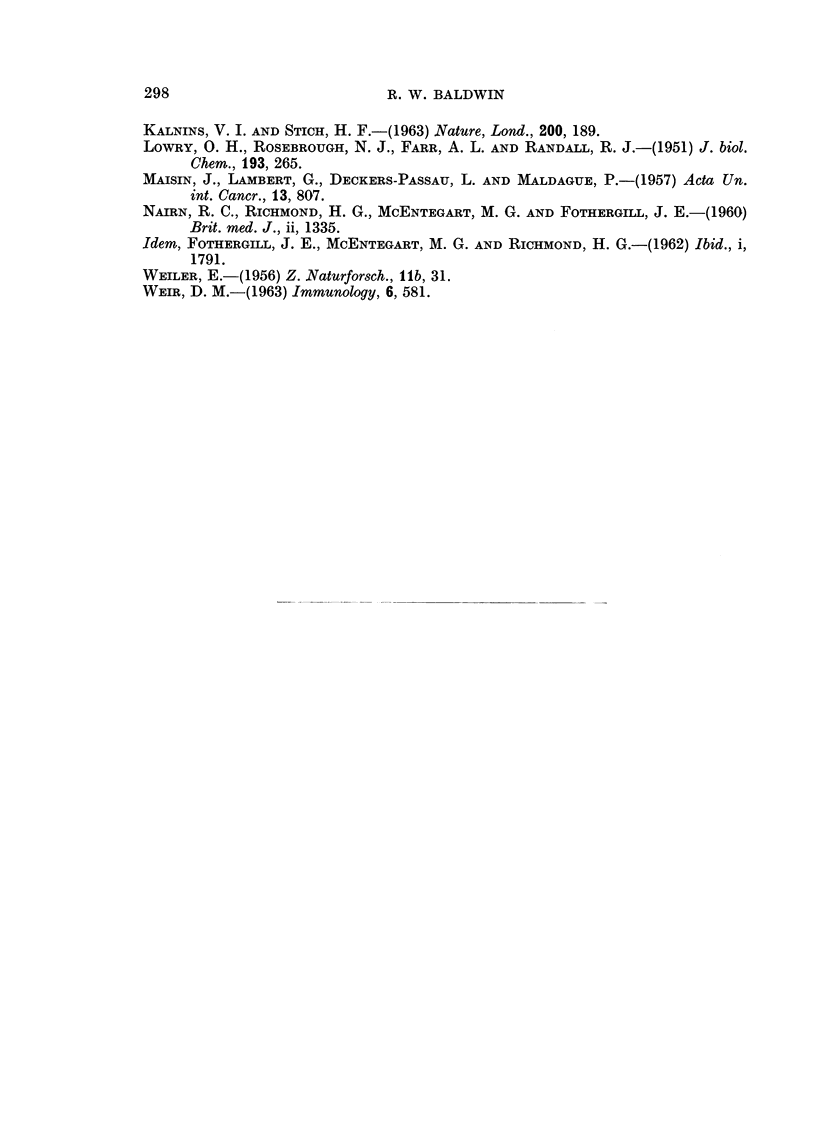# Modification of Cell Antigens During Aminoazo Dye Carcinogenesis in Rat Liver

**DOI:** 10.1038/bjc.1964.33

**Published:** 1964-06

**Authors:** R. W. Baldwin

## Abstract

**Images:**


### MODIFICATION OF CELL ANTIGENS DURING AMINOAZO DYE CARCINOGENESIS IN RAT LIVER

R. W. BALDWIN

*From the Cancer Research Laboratory, The University, Nottingham*

Received for publication May 1, 1964

EVIDENCE has been presented in recent studies indicating that normal tissue antigens are deleted as a result of neoplastic change. Whilst antigenic loss has been demonstrated in both human (Goudie and McCallum, 1962 ; Nairn *et al.*, 1962) and experimental tumours (Barch, 1962 ; Hiramoto, Yagi and Pressman, 1959), the evidence is probably most conclusive from studies with carcinogen induced liver tumours in rats and mice. Hence Weiler (1956) and Nairn *et al.* (1960) using immunohistological procedures have shown that organ specific antigen of normal rat liver was deleted from tumours induced with 4-dimethylaminoazobenzene (DMAB). Similarly, deletion of liver antigen from a transplanted mouse hepatoma has been reported (Engelhardt, Khramkova and Postnikova, 1963) whilst Hiramoto, Bernecky, Jurandowski and Pressman (1961) demonstrated loss of liver microsomal antigen from several transplanted rat hepatomas as well as 2-acetylaminofluorene induced tumours.

Antigen deletion has also been demonstrated recently by direct immunochemical analysis of tissue fractions. Hence in a series of detailed investigations, Abelev and his associates (Abelev *et al.*, 1959 ; Abelev, Khramkova and Postnikova, 1962) have demonstrated deletion of organ specific antigens from transplanted mouse hepatomas originally induced with *o*-aminoazotoluene. Furthermore, Kalnins and Stich (1963) and Deckers (1963) have shown loss of liver antigen from primary and transplanted rat liver tumours induced with DMAB. There is thus now considerable evidence indicating that normal liver antigens are lost from aminoazo dye induced tumour. The studies so far reported, however, have dealt almost exclusively with changes in microsomal antigens whilst as yet little attempt has been made to characterize the components that are deleted from tumour.

In considering the significance of antigenic modification in carcinogenesis, it is important that the total changes in cell antigen population be clearly defined. Accordingly in the present investigations, modifications in the antigenic composition of both microsome and cell sap fractions of rat liver have been determined and partial characterization of the deleted antigens has been obtained by immunoelectrophoresis. Moreover, these changes have been examined using primary tumours induced with DMAB, since it is well established from studies on immunity to transplanted tumours that antigenic modification may occur during transplantation. A preliminary report of these findings has been published (Baldwin, 1963).

### MATERIALS AND METHODS

*Normal rat liver*

Normal liver was taken from 3–4 month old male rats of an inbred Wistar strain which were maintained on a standard cubed diet with water *ad libitum.*

*Aminoazo dye-induced liver tumour*

Male Wistar rats, initially 3 months old, were maintained on an unpolished rice-carrot diet containing 0·06 per cent DMAB for 3 months, and then on the basal rice diet until tumours developed (4–5 months). Additionally, tumours were induced with 3′-methyl-DMAB following continuous administration of the carcinogen at a concentration of 0·075 per cent in a 20 per cent protein diet (Griffin, Nye, Noda and Luck, 1948) for at least 5 months.

In selecting tissue for study, only well defined, non-necrotic tumour masses were taken. These were removed following liver perfusion with cold saline and sucrose solutions, care being taken to exclude gross contamination with liver. Representative parts of each tumour were fixed in 10 per cent formol saline for histological examination and the remainder then treated as described later.

Histological examination of tumour sections, kindly undertaken by Professor G. J. Cunningham, Royal College of Surgeons, London, showed that the tumours could be broadly classified into two main types depending upon cellular arrangements. The first type, a typical example of which is illustrated in Fig. 1, was classified as bile duct carcinoma. In this type of tumour, the main feature was that of pleomorphic acinar proliferation which was lined in parts by a columnar type of epithelium, although in general, most of the cells were flattened in shape. Mitotic figures were numerous. Many of the acini contained necrotic cellular debris, but a few contained mucoid material.

The second type showed solid cords of cells with large nuclei and many mitotic figures often with surrounding zones of necrosis and polymorph infiltration (Fig. 2). Some of these tumours displayed a tendency to acinar formation but this often was localized and when the main mass of tumour was composed of tightly arranged cells, they were classified as hepatocellular carcinoma.

In some individual tumours, precise classification into these cell types was difficult, although in most instances, these two histological appearances were well defined.

When transplanted subcutaneously into isogenic hosts, the DMAB-induced tumours grew in all cases. First generation transplants often grew slowly reaching a size of approximately 3 × 2 × 2 cm. within 4 to 6 weeks. Thereafter transplants grew more rapidly although in some cases, this led to marked necrosis in the tumours. In selecting transplanted tumour for study, only non-necrotic tumour masses were taken and generally, these were from the 2nd or 3rd generation transplants.

### *Sub-cellular Fractionation of Tissues*

Rats were killed by cervical dislocation and the livers perfused immediately with cold 0·15 M NaCl followed by 0·44 M sucrose. Liver or tumour tissue was then removed and further processing carried out in the cold (2° C.). Following mincing with scissors, tissue samples were homogenized in 0·44 M sucrose (1 to

2 ml./g. wet weight of tissue) in a glass Potter-Elvehjem homogenizer fitted with a motor-driven Perspex pestle operating with a controlled clearance of 0·1 mm. For normal liver and other soft tissue samples, homogenization was achieved with the pestle rotating at 3000 to 5000 rev./minute. With harder tumour tissue, greater rotational speeds up to 10,000 rev./minute were necessary to ensure satisfactory homogenization. In all cases, however, the temperature of the homogenates was carefully controlled and not allowed to rise above 5° C.

Following tissue homogenization, cell fractions were isolated essentially as described by D'Amelio and Perlmann (1960). Hence, mitochondrial supernatant fractions were prepared by centrifugation of tissue homogenates at 20,000 *g* for 30 minutes. These fractions were subsequently re-centrifuged at 105,000 *g* for 120 minutes to sediment microsome fractions. After removal of the top half of the supernatant (cell sap) fractions, the microsome pellets were rinsed in 0·44 M sucrose and re-suspended in this medium. Both the cell sap and microsome fractions were then re-centrifuged at 105,000 *g* for a further 120 minutes. The final microsome pellets were rinsed in 0·25 M sucrose and re-suspended in this medium. Again, only the upper half of each cell sap fraction was collected to minimize contamination with microsomal material.

*Cell sap.*—Normal liver fractions prepared as above contained 15–20 mg. protein/ml. whereas the protein content of tumour fractions was somewhat lower (7–16 mg./ml.). Unless used immediately, fractions were stored at −20° C.

*Microsomes.*—Fractions suspended in 0·25 M sucrose were used directly for immunization. For immunochemical analysis, they were solubilized in the cold by the addition of an amount of sodium deoxycholate (5 per cent in 0·2 M glycylglycine buffer, pH 8·0) equivalent to $\frac{3}{4}$ the weight of microsomal protein. After 20 minutes, the deoxycholate concentration was adjusted to 0·4 per cent by the addition of 0·25 M sucrose so that the final solutions contained approximately 6 mg./ml. of microsomal protein. These fractions were either used directly or were further re-centrifuged at 105,000 *g* for 120 minutes to sediment the ribosomal components. After removal of the upper half of the deoxycholate solubilized fraction, the ribosomal pellet was rinsed in 0·25 M sucrose and re-suspended in this medium. Again, unless used immediately, microsome preparations or subfractions were stored at −20° C.

### *Preparation of Antisera*

### *Antigens*

*Normal liver microsomes.*—Whole microsome fractions re-suspended in 0·25 M sucrose so as to contain 25 to 30 mg./ml. microsomal protein. In practice, approximately 30 g. wet weight of rat liver yielded sufficient microsomes for injection into 4 rabbits.

*Normal liver cell sap.*—Fractions in 0·44 M sucrose and containing 15 to 20 mg. protein/ml. were used.

For immunization, freshly prepared liver fractions were emulsified with equal volumes of Freund's adjuvant (complete) and used immediately. Antisera were prepared by intramuscular injection of the Freund's adjuvant mixtures into adult rabbits. A typical immunization schedule consisted of three or four injections at 3 weekly intervals of 2 ml. Freund's adjuvant mixture ; 1 ml. being administered into each hind leg. Rabbits were bled 2 to 3 weeks after the final injection and sera collected in the usual manner. Merthiolate was added to give a final concentration of 0·01 per cent and antisera were stored at −20° C.

In practice, groups of 4 rabbits were immunized with cell fractions prepared from the pooled liver of 4 rats. The final antisera were either stored individually or pooled after their activity had been assessed by immunodiffusion analysis.

### *Immunochemical Procedures*

Double diffusion analyses were carried out in 1 per cent agar gels in buffered saline (0·15 M NaCl, 0·01 M Na phosphate, pH 7·4) containing 0·2 per cent sodium azide as preservative. Cell sap fractions were tested directly in 0·44 M sucrose. Similarly, analysis of microsomal antigens was carried out with preparations solubilized in 0·4 per cent sodium deoxycholate in 0·25 M sucrose. Diffusion wells were filled once with 0·2 ml. of each reagent and the sealed plates were incubated at 2° C. Under these conditions, precipitation patterns usually were fully developed within 7 to 10 days.

For inhibition studies, agar plates were prepared in which tissue fractions were incorporated into the gel. These gels were prepared by adding the tissue fraction rapidly with continuous gentle stirring to a one per cent agar solution in buffered saline at 45° C. As soon as the tissue fraction was fully dispersed, agar plates were prepared in the usual manner at room temperature and when solidified, transferred to a cold room (2° C.) where all further manipulations were conducted.

### *Immunoelectrophoresis*

Immunoelectrophoresis was carried out on 20 × 12 cm. glass plates coated with a 2 mm. layer of 1 per cent agar in Veronal buffer, pH 8·6, $\mu$ 0·025 (Grabar, 1959). Electrophoretic separations were performed at 2° C. employing a potential gradient of 5 volts/cm. for 3 hours. Under these conditions, a current of approximately 20 mA was required for each plate.

Before analysis, tissue fractions were equilibrated with Veronal buffer, pH 8·6, $\mu$ 0·05 by dialysis against 50 to 100 volumes of buffer at 2° C. for 20 hours. When necessary, precipitates were removed by centrifugation (3,000 *g* for 30 minutes) and the solutions were then mixed with an equal volume of 2 per cent agar in distilled water for insertion into agar gel plates. Following electrophoresis, immunodiffusion patterns were developed in the usual manner (Grabar, 1959) with incubation at 2° C. for 7 to 10 days.

### *Chemical Analyses*

Protein was determined by the Lowry technique (Lowry, Rosebrough, Farr and Randall, 1951) using bovine serum albumin as the primary standard.

### RESULTS

*Cell sap antigens*

Comparison of the agar gel cross-reactions of the soluble cytoplasmic (cell sap) fractions from normal liver (Ncs) and DMAB-induced tumour (Tcs) with rabbit antiserum prepared against normal liver cell sap (anti-Ncs) indicates two main features (Fig. 3, top pattern).

1. Two normal liver cell sap antigens do not cross-react with components in the tumour fraction.

2. One tumour antigen does not cross-react with normal liver. Since, however, the antiserum was prepared against normal liver cell sap, this tumour component cannot be an abnormal antigen but must be a normal liver antigen present at a greatly increased concentration. In order to confirm the nature of this component, aliquots of the anti-normal liver cell sap antiserum were pre-absorbed at 4° C. for 4 days with varying amounts of normal liver cell sap and then analysed by agar gel diffusion. Following absorption with normal liver cell sap at a concentration of 8 mg. protein/ml. antiserum, there was no reaction with this fraction although a sharp precipitation line was still detectable following interaction with tumour cell sap. Complete removal of the tumour reaction was obtained when antiserum was absorbed with a larger amount of normal liver cell sap (32 mg. protein/ml. antiserum). These results demonstrate that the apparently abnormal component in tumour is a normal liver antigen. However, consideration of the amount of normal liver cell sap necessary to absorb antibody cross-reacting with tumour indicates that the concentration of this normal liver antigen must be elevated at least 4-fold in tumour.

In all, cell sap fractions from 17 DMAB-induced tumours have been analysed using 6 individual and 2 pooled preparations of rabbit anti-normal liver cell sap antiserum. Loss of normal liver cell sap antigen was observed in all tumour preparations. Usually two well defined normal liver antigens were deleted from tumour (Fig. 3, top pattern), although in certain cases, the number of antigens deleted varied from one to four Additionally, cell sap fractions from tumours induced in 4 rats with 3′-Me-DMAB were analysed. These results again demonstrated that two normal liver cell sap antigens were deleted from tumour.

In order to show that the deletion of normal liver cell sap antigens from DMAB-induced tumour was not due to non-specific dietary effects during carcinogen feeding, in a number of analyses, the tumour cell sap fraction was also cross-reacted with a similar fraction prepared from apparently healthy liver taken from the tumour bearing rat. A typical precipitation pattern obtained with these fractions and anti-normal liver cell sap antiserum is shown in Fig. 3 (lower pattern). This again indicates that two antigenic components detectable in the apparently normal liver fraction (NTLics) do not cross-react with tumour (Tcs). Furthermore, no significant differences were detectable when normal liver cell sap and fractions from apparently healthy liver tissue taken from tumour bearing rats were cross-reacted with anti-normal liver cell sap antiserum.

*Absorption studies*

In order to ascertain whether the observed loss of liver cell sap antigens from DMAB-induced tumour was due simply to a decrease in the concentration of the normal liver components, attempts were made to remove antibody reacting with normal liver cell sap by pre-absorption of antiserum with tumour cell sap. Accordingly, aliquots of antiserum were incubated at 4° C. for 4 days with amounts of tumour cell sap protein ranging from 2 to 20 mg./ml. of antiserum. After removal of precipitates by centrifugation, the absorbed antisera were tested against normal liver and tumour cell sap fractions utilizing the agar gel double diffusion technique. Although complete removal of antibody reacting with tumour cell sap was not easily achieved, following pre-absorption of antiserum with 4 mg./ml. of tumour protein, only one strong precipitation line together with a number of weak lines were formed against this fraction. This antiserum still reacted strongly with normal liver and several well defined precipitation lines were detectable. Following absorption of antiserum with increasing amounts of tumour cell sap, the reaction detectable against this fraction was further diminished. Hence when treated with 16 mg. tumour cell sap/ml. of serum, only a weak reaction was detectable with this fraction (Fig. 4). In contrast, the absorbed serum still reacted as strongly as the unabsorbed serum with normal liver cell sap and at least six precipitation lines were detectable (Fig. 4).

Further evidence indicating that normal liver cell sap antigens are deleted from tumour was obtained when the absorption of antiserum was carried out indirectly by conducting the immunodiffusion analyses in agar gels into which tumour cell sap had been incorporated. Providing that the agar contains a sufficient concentration of tumour cell sap, antibody reacting with antigens in this fraction is inhibited around the antiserum well, whilst non-reacting antibody can diffuse out into the gel and thus is available for reaction.

The cross-reaction of normal liver and tumour cell sap fractions with an anti-normal liver cell sap antiserum in an agar gel containing 2·4 mg./ml. of tumour cell sap protein is illustrated in Fig. 5. With these conditions, all the antibody reacting with tumour cell sap antigens has been inhibited around the antiserum well. Two well defined precipitation lines were still formed, however, following reaction with normal liver cell sap and both showed no cross-reaction with tumour. Clearly therefore, these lines represent normal liver antigens that are absent from tumour. In contrast incorporation of normal liver cell sap into agar gels at a concentration of 1·4 mg. protein/ml. completely abolished the reaction of this fraction with the homologous antiserum.

*Immunoelectrophoretic analysis*

Immunoelectrophoresis allowed further resolution of normal liver cell sap antigens and at least 20 components were detectable following interaction with the homologous antiserum (Fig. 6). Comparison of the immunoelectrophoretic pattern obtained with tumour cell sap clearly indicates that a number of normal liver antigens are not detectable in tumour (Fig. 6) although the complexity of the patterns made identification of specific lines difficult. Immunoelectrophoretic patterns were greatly simplified however when antisera were exhaustively pre-absorbed with tumour cell sap. This is illustrated in Fig. 7 which shows the immunoelectrophoretic patterns obtained with normal liver and tumour cell sap fractions following interaction with anti-normal liver cell sap antiserum before and absorption with tumour cell sap. The precipitation pattern obtained following reaction of normal liver cell sap with the homologous antiserum is again highly complex although clearly, a number of these precipitation lines are not formed following reaction with the tumour fraction. Consideration of the reactions obtained with antiserum pre-absorbed with tumour cell sap (centre well) shows that nine precipitation lines were detectable in normal liver cell sap. Since only a single weak precipitation line was formed following interaction with tumour cell sap, this indicates that at least eight normal liver cell sap antigens were missing from the tumour fraction. Altogether, 6 DMAB-induced tumours have been analysed immunoelectrophoretically using tumour absorbed anti-normal liver cell sap antiserum. These analyses showed that between 5 and 8 normal liver cell sap antigens were deleted from tumour.

*Microsomal antigens*

Although rabbit antisera were prepared against whole normal liver microsomes, for immunochemical analysis, microsomal preparations were solubilized in 0·4 per cent sodium deoxycholate. This treatment, which generally is supposed to solubilize lipoproteins of the membranes of the endoplasmic reticulum liberating the contents of the membranous vesicles, has been claimed (D'Amelio and Perlmann, 1960) to be without great effect on the properties of microsomal antigens. Because of the low yield of microsomes from DMAB-induced liver tumour (1–2 mg./g. wet weight of tissue), these deoxycholate solubilized fractions were not generally sub-fractionated into the soluble and ribosomal components. However, preliminary studies demonstrated that deoxycholate solubilization yielded fractions in which the antigenic composition was reasonably reproducible. Moreover, as shown in Fig. 8, the precipitation pattern obtained with whole solubilized normal liver microsome fractions represented mainly interactions of the soluble proteins (Sp) and little or no reaction was produced by either the ribosomal (Rp) or membranous (Mm) components at the concentrations contained in the whole fraction.

A typical cross-reaction pattern obtained following agar gel double diffusion analysis of deoxycholate solubilized microsome fractions of normal liver (N mic) and DMAB-induced tumour (T mic) with anti-normal liver microsome antiserum (anti-N mic) is shown in Fig. 9 (top pattern). This demonstrates quite clearly that two major antigenic components of normal liver microsomes do not cross-react with tumour. Additionally, a third poorly defined precipitation band formed near to the well containing normal liver microsomes does not show any obvious cross-reaction with tumour. In contrast, no differences were detectable in the cross-reaction pattern obtained with a microsome fraction from normal liver and a similar fraction prepared from apparently healthy liver tissue taken from the tumour bearing rat (N TLi mic). Thus, as shown in Fig. 9 (lower pattern), the two major and one weaker lines which were not formed against tumour were detectable in both liver fractions. This demonstrates that the deletion of normal liver microsomal antigen observed in DMAB-induced tumour was not due to non-specific effects during carcinogen administration.

In all, the antigenic composition of 15 tumour microsomal fractions, each of which was prepared from pooled tumour tissue taken from 2 to 4 rats, have been compared with that of normal liver microsomes using 5 individual and one pooled sample (4 rabbits) of anti-normal liver microsome antiserum. Loss of normal liver microsomal antigen from tumour was observed in all the analyses, although the number of components deleted showed some variation. This most probably was due to poor resolution of deoxycholate solubilized microsomal components by simple diffusion. Hence in a number of analyses, the normal liver component not cross-reacting with tumour was detected as a single dense precipitation line and this most likely represented poor resolution of the two normal liver lines shown in Fig. 9 (top pattern).

Loss of normal liver microsomal antigen was also observed in two tumours induced with 3′-methyl-DMAB. Moreover, it was shown that transplants of one DMAB-induced tumour were also deficient in certain normal liver microsomal antigens. This is illustrated in Fig. 10 which shows the cross-reaction pattern of microsome fractions from normal liver (N mic) and from a 3rd generation transplant of a DMAB-induced tumour (T/3 mic) with anti-normal liver microsome antiserum. Thus, as with the primary DMAB-induced tumour, two major precipitation lines together with a third more diffuse line formed with normal liver do not cross-react with components in the transplanted tumour.

---

### EXPLANATION OF PLATES

FIG. 1.—Section of liver tumour showing pleomorphic acinar proliferation. The lining cells vary from columnar to flat cuboidal in type. Most of the acini contain necrotic cellular debris, but a few contain mucoid material. There are areas of necrosis in some parts of the tumour.

FIG. 2.—Section of liver tumour consisting of solid cords of cells which have large nuclei, many showing mitotic figures. There are surrounding zones of necrosis and polymorph infiltration but very little evidence of acinar formation.

FIG. 3.—Agar gel precipitation reaction of tissue cell sap fractions with antiserum prepared against normal rat liver cell sap (anti-N.cs).

Ncs—Normal liver cell sap.

Tcs—DMAB-induced tumour cell sap.

NTLics—Cell sap fraction of liver taken from tumour bearing rat.

All cell sap fractions contained 13·7 mg. protein/ml.

FIG. 4.—Cross-reaction in agar of normal liver and tumour cell sap fractions with anti-normal liver cell sap antiserum pre-absorbed with tumour cell sap (16 mg. protein/ml.). See Fig. 3 for legends. Protein content of cell sap fractions, 11 mg./ml.

FIG. 5.—Precipitation reactions of normal liver and tumour cell sap fractions (protein content, 11 mg./ml.) with anti-normal liver cell sap antiserum in agar gel containing tumour cell sap (2·4 mg. protein/ml.). See Fig. 3 for legends.

FIG. 6.—Immunoelectrophoresis of normal liver and tumour cell sap fractions (protein content, 8–9 mg./ml.). See Fig. 3 for legends.

FIG. 7.—Comparison of the immunoelectrophoretic patterns obtained following reaction of cell sap fractions with anti-normal liver cell sap antiserum (anti Ncs) before and after absorption with tumour cell sap (Tcs).

FIG. 8.—Agar gel precipitation reactions of deoxycholate solubilized normal liver microsomes and sub-fractions with anti-normal liver microsome antiserum (anti Nmic).

*Mic.*—Whole microsome fraction solubilized in 0·4 per cent sodium deoxycholate (6 mg. protein/ml.).

Sp.—Soluble fraction of whole microsomes in 0·4 per cent deoxycholate (5 mg. protein/ml.).

Rp.—Ribonucleoprotein fraction re-suspended in 0·25 M sucrose at a concentration equivalent to that in the whole microsome fraction (0·4 mg. protein/ml.).

MM.—Fluffy layer isolated from above Rp pellet on centrifugation of deoxycholate solubilized microsomes and re-suspended in 0·25 M sucrose at its original concentration (0·7 mg. protein/ml.).

FIG. 9.—Agar gel precipitation patterns of deoxycholate solubilized microsome fractions from normal liver and DMAB-induced tumour with anti-normal liver microsome antiserum (anti-N mic).

N mic—Normal liver microsomes.

T mic—DMAB-tumour microsomes.

N.T.Li mic—Microsome fraction of liver taken from tumour bearing rat.

Protein content of microsome fractions, 6–7 mg./ml.

FIG. 10.—Agar gel cross-reaction pattern of deoxycholate solubilized microsome fractions of normal liver and a 3rd generation transplant of a DMAB-induced liver tumour with anti-normal microsome antiserum (anti N mic).

N mic—Normal liver microsome, DOC solubilized (6·7 mg. protein/ml.).

T/3 mic—Microsome fraction from 3rd generation transplant of DMAB-induced tumour, DOC solubilized (6 mg. protein/ml.).

FIG. 11.—Immunoelectrophoresis of deoxycholate solubilized microsome fractions of normal liver and DMAB-induced tumour.

Normal liver—N mic (Doc sol.) (3·2 mg. protein/ml.).

DMAB-tumour—T mic (Doc. sol.) (3·0 mg. protein/ml.).

Antiserum—anti-normal liver microsome (anti-N mic).

FIG. 12.—Immunoelectrophoresis of microsome fractions utilizing anti-normal liver microsome antiserum pre-absorbed with tumour microsomes (see Fig. 11 for legends).

Protein content of microsome fractions 5–6 mg./ml.

1

2

Baldwin.

Ncs
anti-Ncs
Tcs
NTLi cs

3

Tcs
Ncs
anti-Ncs (abs.)

4

Tcs anti-Ncs

Ncs

6

Anti-Ncs

Ncs

Tcs

5

Anti NCs
Tcs
Anti-Ncs(abs. with Tcs)
Ncs
Anti-Ncs

7

Mic
Sp
Nmic
Mm
Rp

8

Nmic
Tmic
anti-
Nmic
NTLi
mic
Nmic

9

Nmic
T/3
mic
anti-
Nmic
T/3
mic

10

Anti-Nmic

Tmic(Doc sol)

11

Tmic(Doc sol)

Anti-N mic(abs with Tmic)

N mic(Doc sol)

12

*Absorption studies*

In order to confirm that normal liver microsomal antigens were deleted from DMAB-induced tumour, attempts were made to remove antibody reacting with these liver antigens by pre-absorption of antiserum with tumour microsomes. Thus aliquots of an anti-normal liver microsome antiserum were treated at 4° C. for 4 days with varying amounts of tumour microsomal protein. After removal of the precipitates by centrifugation, the absorbed sera were then tested against normal liver and tumour microsome fractions using the agar gel diffusion technique. As shown in Table I, absorption with tumour microsomes at a concentration

TABLE I.—*Absorption of Anti-Normal Liver Microsome Antiserum with Deoxycholate Solubilized Tumour Microsomes*

| Number of precipitation lines formed following reaction with | Absorption conditions : Amount of tumour microsomal protein. mg./ml. antiserum | | | | |
|---|---|---|---|---|---|
| | 0 | 1·5 | 3·0 | 4·5 | 6·0 |
| Normal liver microsomes . | 5 | 3 | 2 | 2 | 2+1 weak |
| Tumour microsomes . . | 2–3 | 1 | 0 | 0 | 1 weak |

of 1·5 mg./ml. of antiserum almost completely removed antibody reacting with this fraction and only a single weak line was detectable. This reaction line was completely removed following absorption with 3 mg. tumour microsomal protein/ml. antiserum and in this case it was shown also that the absorbed antiserum contained slight excess of tumour antigens. Two strong precipitation lines were still detectable following interaction with a normal liver microsome fraction. These lines were unaffected even when the antiserum was absorbed with up to 6 mg./ml. of tumour microsome protein. In this case, however, a third weak component was detectable in both normal liver and tumour fractions. This most probably was due to non-precipitation of some antigen-antibody complexes during absorption because of great excess of absorbing antigen.

*Immunoelectrophoretic analysis*

For immunoelectrophoresis, it was necessary to equilibrate deoxycholate solubilized microsome fractions against Veronal buffer, pH 8·0, $\mu$, 0·05. However, little or no precipitate was formed following dialysis of fractions for 20 hours at 2° C. against 50 to 100 volumes of buffer and the final samples were analysed at a concentration of 3 to 7 mg./ml. of microsomal protein. Immunoelectrophoresis was carried out for 3 hours with a potential gradient of approximately 5 volts/cm. Under these conditions, the major antigenic fraction migrated 2–3 cm. towards the cathode and as shown in Fig. 11 permitted the detection of at least 10 components reacting with anti-normal liver microsome antiserum. Precise evaluation of the number of components was difficult since a band containing the major antigenic fraction was not well resolved. However, compared with the precipitation pattern obtained with the tumour microsome fraction (Fig. 11), it is obvious that a number of antigenic components detectable in the major band are deleted from tumour.

In order to further define the normal liver microsomal antigens that are deleted from tumour, immunoelectrophoretic analyses were also carried out using anti-normal liver microsome antiserum which had been exhaustively pre-absorbed with a deoxycholate solubilized tumour microsome fraction. As shown in Fig. 12 this antiserum no longer reacted with the tumour microsome fraction. In contrast, a major band containing at least 3 components and two further well defined lines were formed following interaction with the normal liver microsome fraction. Additionally a group of at least four poorly resolved lines were detectable close to the well containing antigen. Hence whilst the conditions of electrophoresis are not entirely satisfactory for characterization of liver microsomal antigens, this procedure permits the detection of at least 10 normal liver microsomal antigens which are deleted from DMAB-induced tumour.

### DISCUSSION

Loss of normal liver microsomal antigen from DMAB-induced tumour has been demonstrated by Weiler (1956) and Nairn *et al.* (1960) using immunohistological procedures. Similarly Hiramoto *et al.* (1961) showed that liver tumours induced with 2-acetylaminofluorene did not stain with antisera prepared against normal liver microsomes. That normal liver microsomal antigens are deleted from DMAB-induced tumour has been confirmed in the present studies whilst application of immunodiffusion techniques has allowed some characterization of the antigens involved. Direct cross-reaction of deoxycholate solubilized microsome fractions with anti-normal liver microsome antiserum also indicated that there are a number of antigenic components common to both normal liver and tumour. This is hardly surprising considering that liver microsomes probably contain many antigenic structures and it is unlikely that all of these would be deleted or modified following neoplastic change. These results indicate, however, serious limitations in the application of the immunohistological technique for assessing antigenic loss, since previous studies failed to detect these weak common antigens.

Whilst direct agar gel diffusion studies indicated that certain components in deoxycholate solubilized fractions of tumour microsomes did not cross-react with normal liver it is possible that this was due to quantitative rather than qualitative modifications. However, antibody reacting with the normal liver antigens which were not detectable in tumour could not be removed by pre-absorption of anti-serum with tumour microsomes. Hence, although the possibility that trace amounts of normal liver microsomal antigens are present cannot be excluded, it is considered that the loss of reaction with tumour represents antigen deletion.

Antigen deletion in DMAB-induced tumour was not limited to microsomal components alone, and significant losses were observed also in the cell sap antigens (Fig. 3). Again absorption studies demonstrated that antigen deletion in tumour was not due simply to quantitative losses of the normal liver components. Additionally immunoelectrophoretic analysis of normal liver cell sap fractions using homologous antiserum pre-absorbed with tumour allowed partial characterization of the antigens involved and indicated that at least five components are deleted from tumour. Clearly therefore, a complex series of cell antigen changes occurs during aminoazo dye carcinogenesis in rat liver resulting in the deletion of liver microsomal and cell sap antigens from tumour. Additionally, the concentration of one normal liver cell sap antigen was increased at least 4-fold in tumour.

Loss of liver antigen from DMAB-induced hepatoma has been reported also by Kalnins and Stich (1963) although since antisera were prepared against whole liver homogenates, identification of the deleted antigens was not possible. It was reported, however, that individual hepatomas differed in the number of antigens lost. Deckers (1963) has also demonstrated that liver microsomal antigen was deleted from a transplanted rat hepatoma, whilst Friedrich-Freksa (1963) has partially purified and characterized one of the liver microsomal antigens that are missing from DMAB-induced tumour. These findings, together with the extensive investigations of Abelev and co-workers using *o*-aminoazotoluene induced hepatomas in mice (Abelev, Avenirova, Engelhardt, Baydakova and Stepanchemok-Rudnik, 1959 ; Abelev, Khramkova and Postnikova, 1962 ; Abelev, 1963) thus present an extensive body of evidence demonstrating that cell antigens are deleted from aminoazo dye induced liver tumour.

In considering the significance of the loss of normal liver cell antigens from DMAB-induced tumour, the possibility needs to be considered that the changes simply reflect alterations in cell populations. Thus liver contains at least three types of cells each of which may possess its own antigenic characteristics. In consequence antigen deletion may not be due to alterations in the antigenic composition of the neoplastic cell, but might for instance be due to gross alterations in the proportions of normal cells.

A common form of neoplasm encountered in the present investigation has been that of bile duct origin. Hence it may be argued that with these tumours, loss of antigens was due to replacement of normal liver cells by bile duct cells. However, if this were so, gradual deletion of cell antigens should occur during the earlier stages of aminoazo dye carcinogenesis when there is considerable proliferation of bile duct cells (Daoust and Cantero, 1959) with concomitant losses of hepatic enzymes (Jones, 1963), but such changes have not been observed. Moreover studies on the proportion of various cell types in liver during DMAB carcinogenesis (Daoust and Cantero, 1959) indicate that these variations are probably not sufficient to account for the observed loss of cell antigens in tumour. Other tumours used in these studies showed the histological characteristics of true hepatocarcinoma and significantly, these also proved to be deficient in certain normal liver antigens. In view of the wide diversity of neoplastic cell types, it is perhaps surprising that the differences observed between the antigenic composition of normal and tumour cell fractions was so reproducible. It is possible however that the procedures used for resolving tissue antigens were imprecise and individual differences may be detected with more refined techniques. Moreover, the requirement for relatively large samples of tumour tissue results in an increased complexity of types of cells involved, so that individual differences between tumours may be obscured. Hence a more critical assessment of the significance of cell antigen changes in DMAB-carcinogenesis should be obtained from immunohistological studies using monospecific antisera against purified normal liver antigens which the present studies indicate to be deleted from tumour. Additionally however, the possibility that liver cell and bile duct tumours have common antigenic structures needs to be considered, particularly in view of the suggestion that such tumours have a common origin in a primitive hepatic cell (Maisin, Lambert, Deckers-Passau, and Maldague, 1957).

In order to ascertain the significance of cell antigen deletions in DMAB carcinogenesis, it is clearly important to discover the physiological role of these antigens. Only then will it be possible to decide whether the antigenic changes are causally connected with the neoplastic transformation or simply are alternative expressions of biochemical change. The major problem however is to devise possible methods for assaying purified tissue antigens since characterization in biochemical terms such as enzyme content may not be relevant. Thus although Friedrich-Freksa (1963) has partially purified a microsomal antigen which is deleted from DMAB-induced tumour, its physiological action is still unknown beyond the finding that it contains glucose-6-phosphatase activity. According to Green (1954) the essential change in the neoplastic cell is the loss of a tissue specific factor (antigen) which is considered to be located in the endoplasmic reticulum. Similarly, Burch (1963) from theoretical considerations of mechanisms of carcinogenesis has postulated that the critical change is the removal or alteration of a microsomal " differentiating factor ". That tissue controlling factors exist is clearly demonstrated in the studies of Bullough (1962) on substances (chalones) which control the mitotic activity of mouse epidermis and so the possibility that the normal liver microsomal antigens deleted from tumour possess growth controlling properties clearly requires further investigation. Whilst generally it has been assumed that modification of microsomal antigen represents the critical deletion in carcinogenesis, the present studies also indicate that liver cell sap antigens are missing from DMAB-induced tumour. The nature and function of these cell sap antigens also requires investigation particularly in view of the suggestion (Burwell, 1963) that soluble tissue factors may be responsible for growth control of many differentiated tissues. Moreover, Bullough (1962) has shown that at least part of the epidermis-specific mitotic inhibitor (chalone) is water soluble.

According to the immunological concept of carcinogenesis, loss of cell antigen in the neoplastic cell occurs as a consequence of an immune response to modified tissue antigens. The finding that abnormal tissue antigens are formed as a result of covalent bonding of a metabolite of DMAB to liver protein (Baldwin, 1962*a*) lends support for such an hypothesis. Moreover it has been shown that liver antigens are liberated and circulate in the blood stream following induction of acute liver damage with nitroso-dimethylamine and other hepatotoxic agents (Baldwin, 1962*b*). Hence it is also likely that the abnormal DMAB-antigens will be liberated during carcinogenesis and so will be available for the induction of an immune response. Whilst such an immune response has still to be demonstrated, it is perhaps relevant that liver autoantibody has been detected in rat serum following induction of liver damage with carbon tetrachloride (Weir, 1963). Possible mechanisms whereby an immune response to modified tissue antigens could effect the neoplastic change are more difficult to conceive. However, Burch (1963) has suggested that the modified cell antigen is a lipoprotein component of the cell membrane and endoplasmic reticulum which normally is the source of one or more factors that combine with or affect repressor molecules to determine the differentiation state of the cell and its overall metabolic pattern. Hence if antibody blocked or deleted such antigenic structures, this could lead to an irreversible change in the resulting neoplastic cell.

Whilst the present findings have been interpreted within the framework of the immunological concept of carcinogenesis, other interpretations are clearly possible. Thus, for example, antigen deletion may simply reflect mutational changes induced by the carcinogen. These interpretations, however, permit the design of further experiments which should allow a critical evaluation of the role of tissue immune reactions in chemical carcinogenesis.

SUMMARY

Modifications in the antigenic composition of sub-cellular fractions of rat liver during aminoazo dye carcinogenesis have been investigated. Normal rat liver cell sap antigens were found to be deleted from DMAB-induced liver tumour and partial characterization of these antigens was obtained by immunoelectrophoresis. Additionally, the concentration of one normal liver cell sap antigen was shown to be increased at least 4-fold in tumour. Loss of normal liver microsomal antigen from DMAB-induced tumour was also demonstrated and the deleted antigens characterized by immunoelectrophoresis.

No differences were detectable between the antigenic composition of cell fractions from normal liver and apparently healthy liver taken from tumour-bearing rats. It was concluded therefore that loss of cell antigens from tumour occurred as a result of neoplastic change rather than from non-specific effects during carcinogen feeding.

The author wishes to thank Professor G. J. Cunningham, Royal College of Surgeons, London, for carrying out the histological studies, and Mrs. M. E. Marshall for skilled technical assistance. This work was supported by a block grant from the British Empire Cancer Campaign.

REFERENCES

ABELEV, G. I.—(1963) *Acta Un. int. Cancr.*, **19**, 80.
*Idem*, AVENIROVA, Z. A., ENGELHARDT, N. V., BAYDAKOVA, Z. L. AND STEPANCHENOK-RUDNIK, G. I.—(1959) *Dokl. Akad. Nauk, SSSR*, **124**, 1328.
*Idem*, KHRAMKOVA, N. I. AND POSTNIKOVA, Z. A.—(1962) *Neoplasma*, **9**, 123.
BALDWIN, R. W.—(1962*a*) *Brit. J. Cancer*, **16**, 749.—(1962*b*) *Rep. Brit. Emp. Cancer Campgn*, **40**, 436.—(1963) *Acta. Un. int. Cancr.*, **19**, 545.
BARCH, S.—(1962) *Exp. Cell Res.*, **27**, 548.
BULLOUGH, W. S.—(1962) *Biol. Rev.*, **37**, 307.
BURCH, P. R. J.—(1963) *Nature, Lond.*, **197**, 1145.
BURWELL, R. G.—(1963) *Lancet*, ii, 69.
D'AMELIO, V. AND PERLMANN, P.—(1960) *Exp. Cell Res.*, **19**, 383.
DAOUST, R. AND CANTERO, A.—(1959) *Cancer Res.*, **19**, 757.
DECKERS, C.—(1963) *Acta Un. int. Cancr.*, **19**, 172.
ENGELHARDT, N. V., KHRAMKOVA, N. I. AND POSTNIKOVA, Z. A.—(1963) *Neoplasma*, **10**, 133.
FRIEDRICH-FREKSA, H.—(1963) ' Cellular Control Mechanisms and Cancer '. Amsterdam (Elsevier). In Press.
GOUDIE, R. B. AND MCCALLUM, H. M.—(1962) *Lancet*, i, 348.
GRABAR, P.—(1959) ' Methods of Biochemical Analysis ', **7**, 1. Interscience (New York).
GREEN, H. N.—(1954) *Brit. med. J.*, ii, 1374.
GRIFFIN, A. C., NYE, W. N., NODA, L. AND LUCK, J. M.—(1948) *J. biol. Chem.*, **176**, 1225.
HIRAMOTO, R., BERNECKY, J., JURANDOWSKI, J. AND PRESSMAN, D.—(1961) *Cancer Res.*, **21**, 1372.
*Idem*, YAGI, Y. AND PRESSMAN, D.—(1959) *Ibid.*, **19**, 874.
JONES, G. R. N.—(1963) *Brit. J. Cancer*, **17**, 153.

KALNINS, V. I. AND STICH, H. F.—(1963) *Nature, Lond.*, **200**, 189.
LOWRY, O. H., ROSEBROUGH, N. J., FARR, A. L. AND RANDALL, R. J.—(1951) *J. biol. Chem.*, **193**, 265.
MAISIN, J., LAMBERT, G., DECKERS-PASSAU, L. AND MALDAGUE, P.—(1957) *Acta Un. int. Cancr.*, **13**, 807.
NAIRN, R. C., RICHMOND, H. G., MCENTEGART, M. G. AND FOTHERGILL, J. E.—(1960) *Brit. med. J.*, ii, 1335.
*Idem*, FOTHERGILL, J. E., MCENTEGART, M. G. AND RICHMOND, H. G.—(1962) *Ibid.*, i, 1791.
WEILER, E.—(1956) *Z. Naturforsch.*, **11***b*, 31.
WEIR, D. M.—(1963) *Immunology*, **6**, 581.